# Supplementary material for: In situ inward epitaxial growth of bulk macroporous single crystals
Source: Nat Commun. 2017 Dec 19;8:2178. doi: 10.1038/s41467-017-02197-6 (PMC5736656; doi:10.1038/s41467-017-02197-6)

Supplementary Figure 1: TEM images tilted along the top-to-bottom direction (near GaN[0002] orientation). Tiltingangles: **(a)** 20, **(b)** 15, **(c)** 10, **(d)** 5, **(e)** 0 (near $[1\bar{1}00]$ zone axis), **(f)**–5, **(g)**–10, **(h)** –15, and **(i)**–20°.

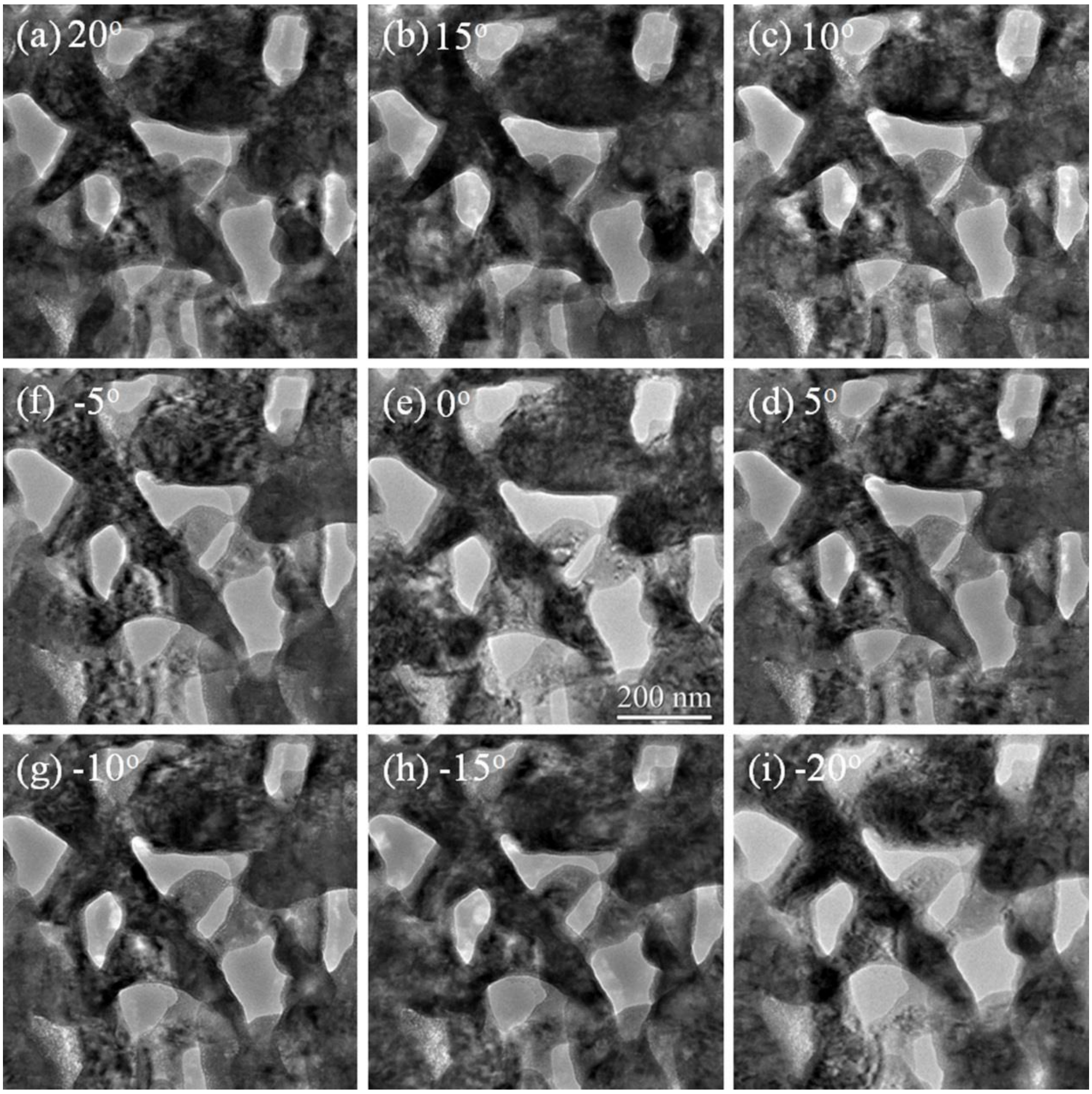

Supplementary Figure 2: Cross-sectional HAADF images with [11$\bar{2}$0] zone axis of a porous GaN film grown inward on a $LiGaO_2$ (001) substrate. Sample thicknesses:**(a)**, **(b)**90 nm; **(c)**, **(d)** 300 nm.

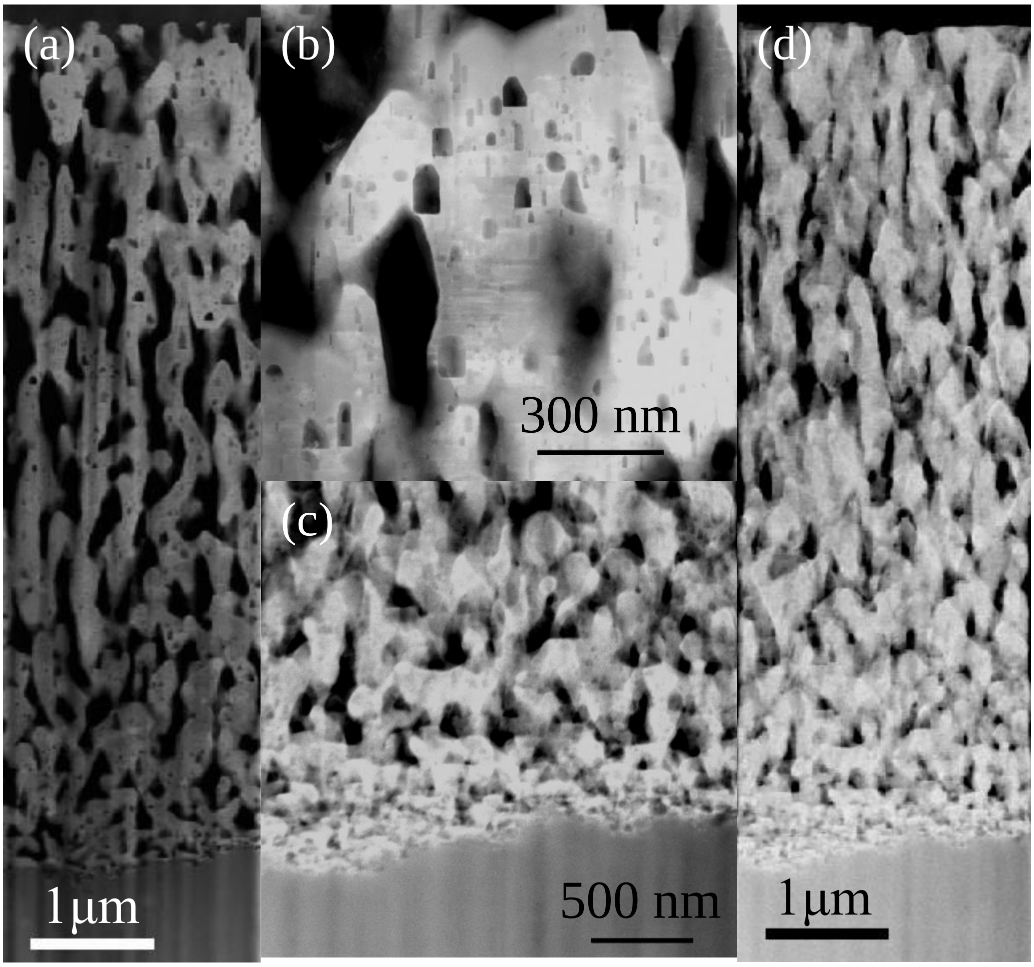

Supplementary Figure 3: Cross-sectional TEM characterizations with [1$\bar{1}$00] zone axis of a porous GaN film grown inward on a $LiGaO_2$ (001) substrate.(a)HAADF image. **(b)**Typical SAED pattern of the porous area. **(c)** SAED pattern at the interface. **(d)**, **(e)** HAADF imagerevealing isosceles triangular shapes of the porous structures. **(f)** Experimental and simulated CBED patterns; the numbers are Miller indices. The results reveal that the top angles of the isosceles triangular porous structures point to [000$\bar{2}$] GaN.

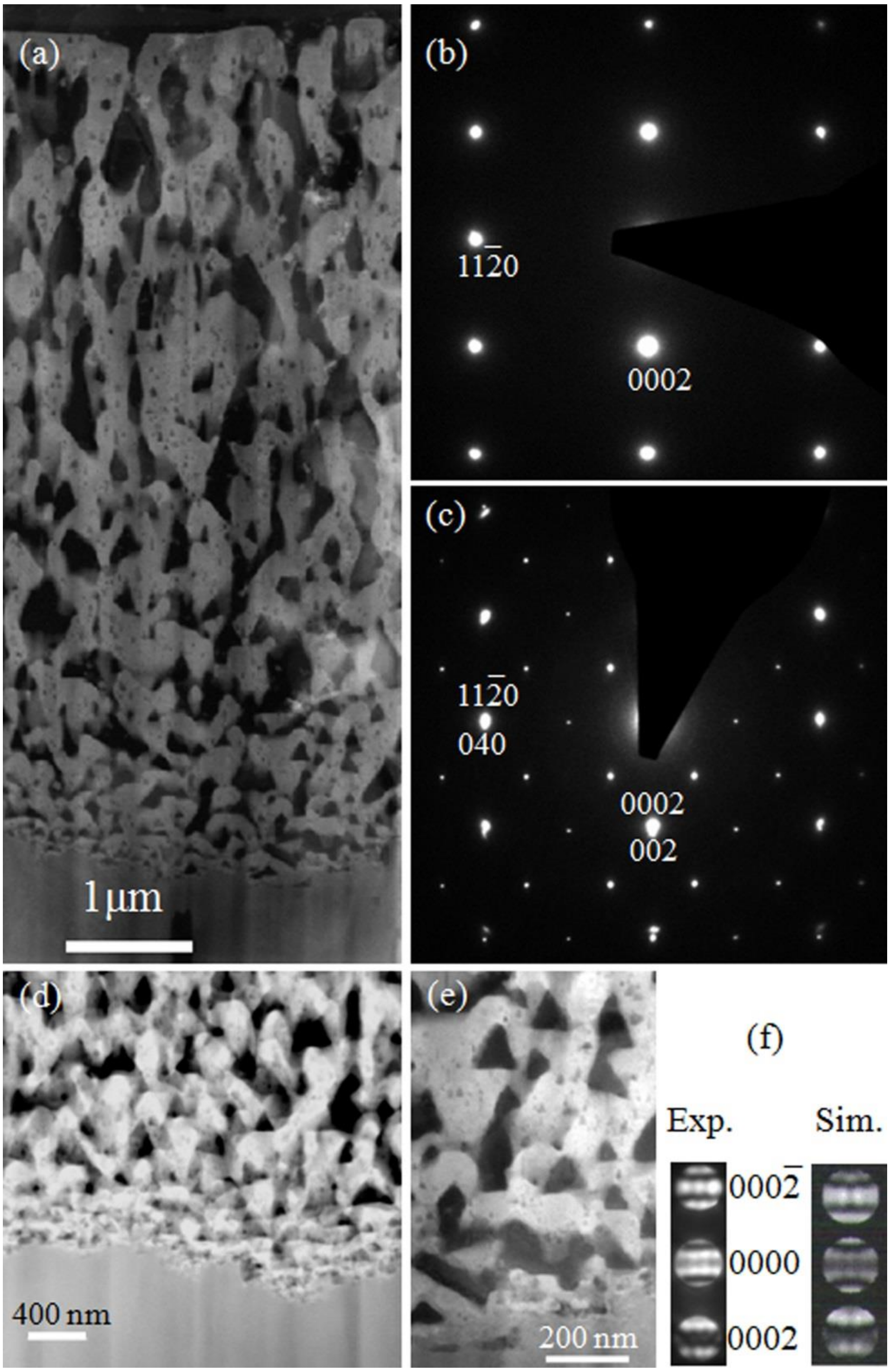

Supplementary Figure 4: High-resolution TEM image of porous GaN film on $LiGaO_2$ (001) substrate at the interface with [1$\bar{1}$00] zone axis.

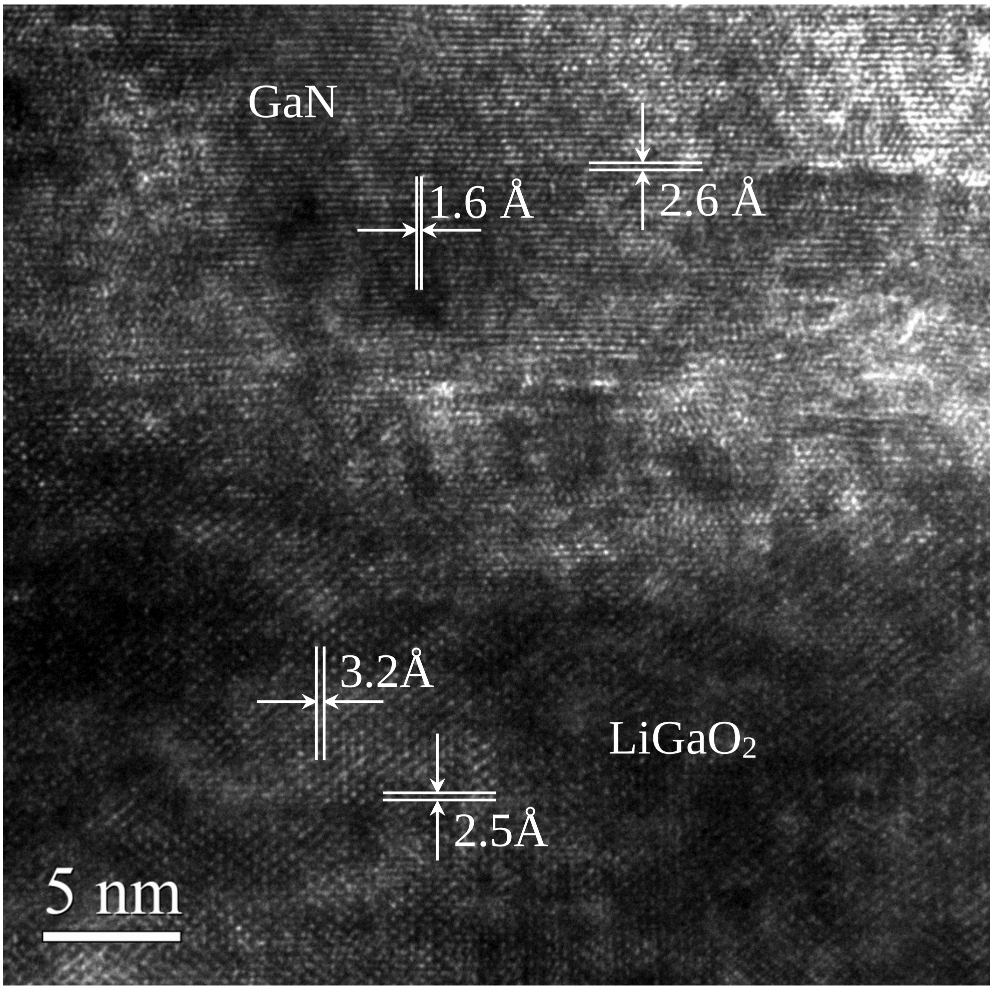

Supplementary Figure 5: In situ reverse epitqxial growth of nonpolar porous GaN crystals. (a) XRD results of m-plane grown along the [10-10] direction on (100) $LiGaO_2$. (b) XRD results of a-plane grown along the [11-20] direction on (010) $LiGaO_2$ substrate.

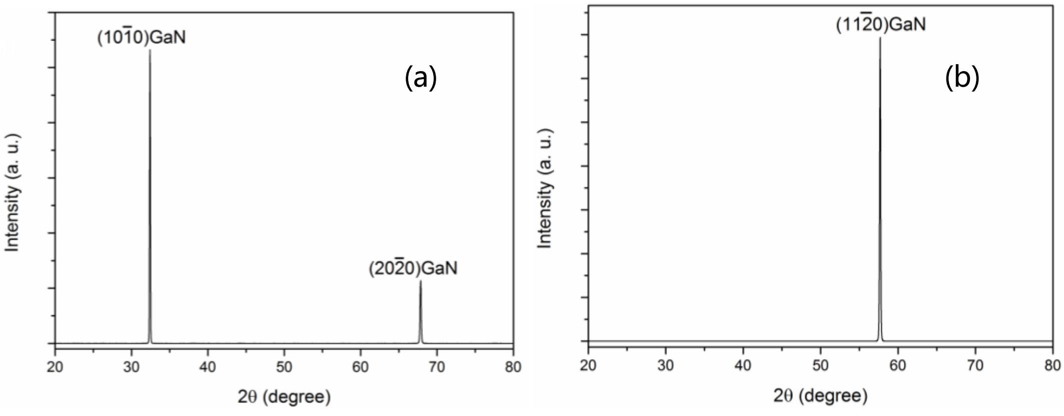

Supplementary Figure 6: XRD patterns of porous GaN crystals grown on $LiGaO_2$ at 267 mbar and a flow rate of 200 sccm of NH3 for 20 hours at different growth temperatures. (a) 750 ^oC. (b) 800 ^oC. (c) 850 ^oC. (d) 900 ^oC. (e) 950 ^oC. (f) 1000 ^oC. (g) 1050 ^oC.

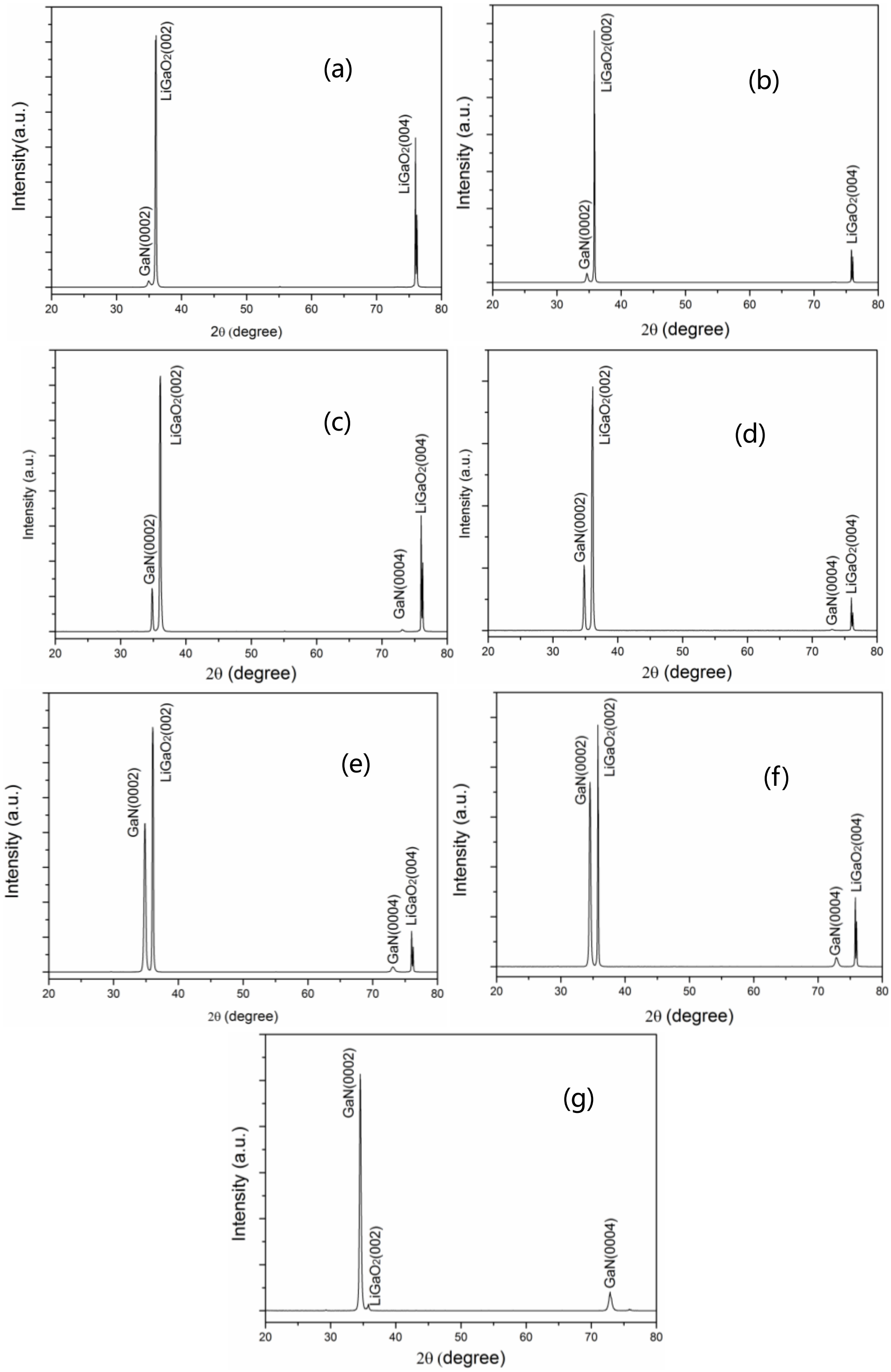

Supplementary Figure 7: SEM images of porous GaN crystals grown on $LiGaO_2$ at 267 mbar and a flow rate of 200 sccm of NH_3 for 20 hours at different growth temperatures. (a) 750 ^{o}C. (b) 800 ^{o}C. (c) 850 ^{o}C. (d) 900 ^{o}C. (e) 950 ^{o}C. (f) 1000 ^{o}C. (g) 1050 ^{o}C.

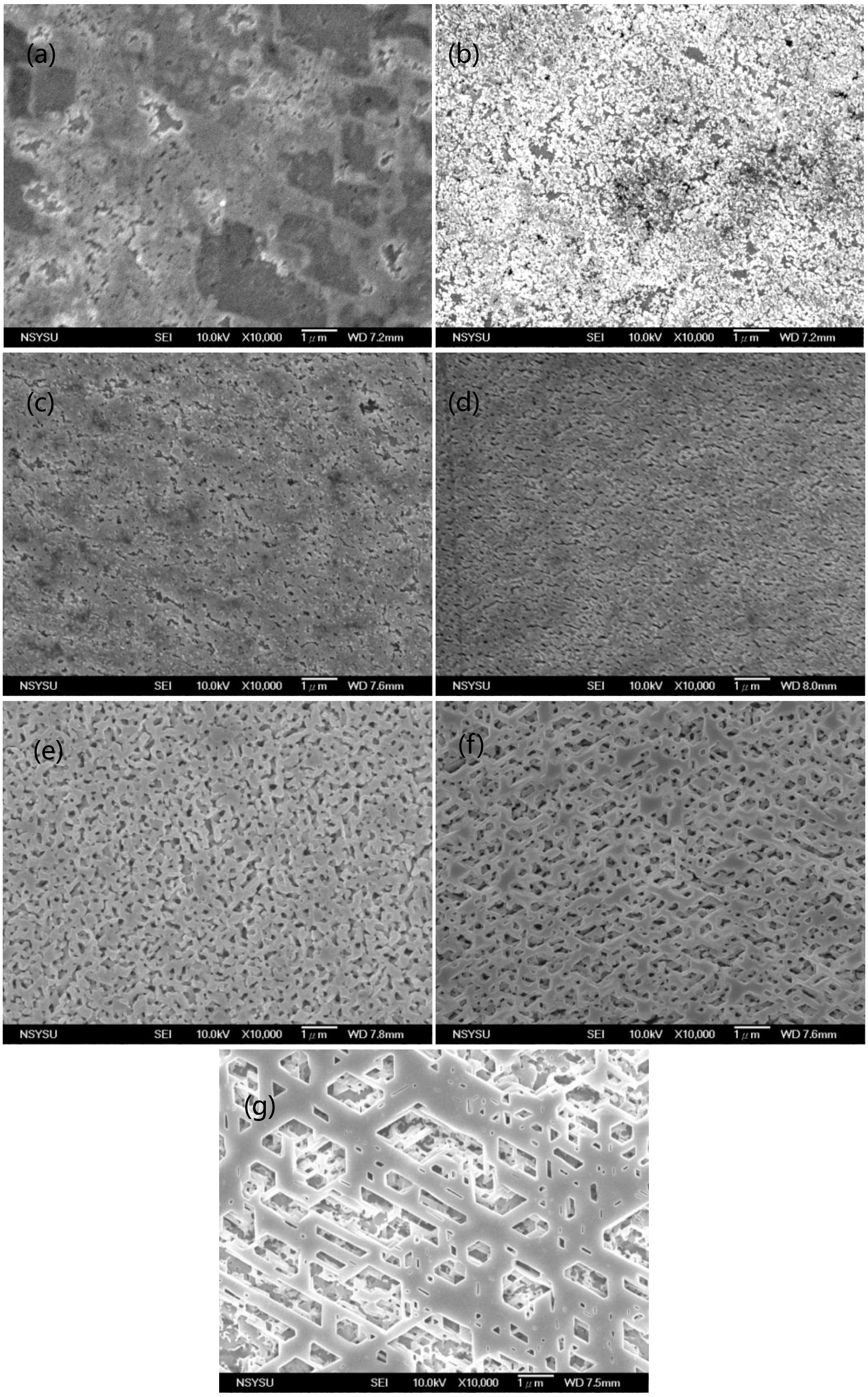

Supplementary Figure 8: FWHM of porous GaN (0002) and (10-10) rocking curves grown on $LiGaO_2$ at 950 °C and a pressure of 267 mbar for 20 hours with different NH_3 flow rate.

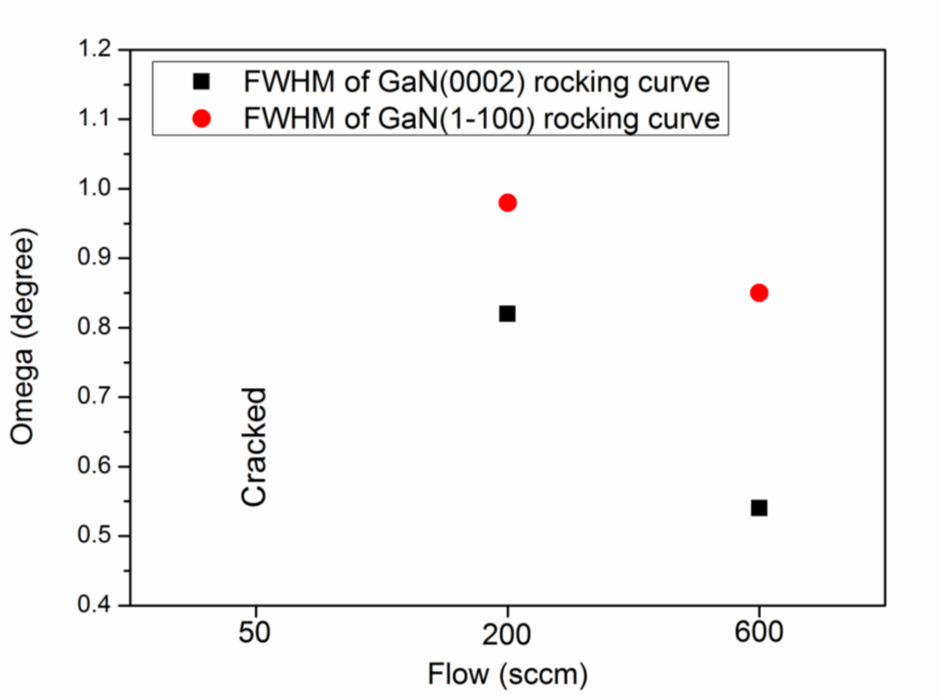

Supplementary Figure 9: FWHM of porous GaN (0002) and (10-10) rocking curves grown on $LiGaO_2$ at 950 ^oC and a NH_3 flow rate of 600 sccm for 20 hours with different growth pressure.

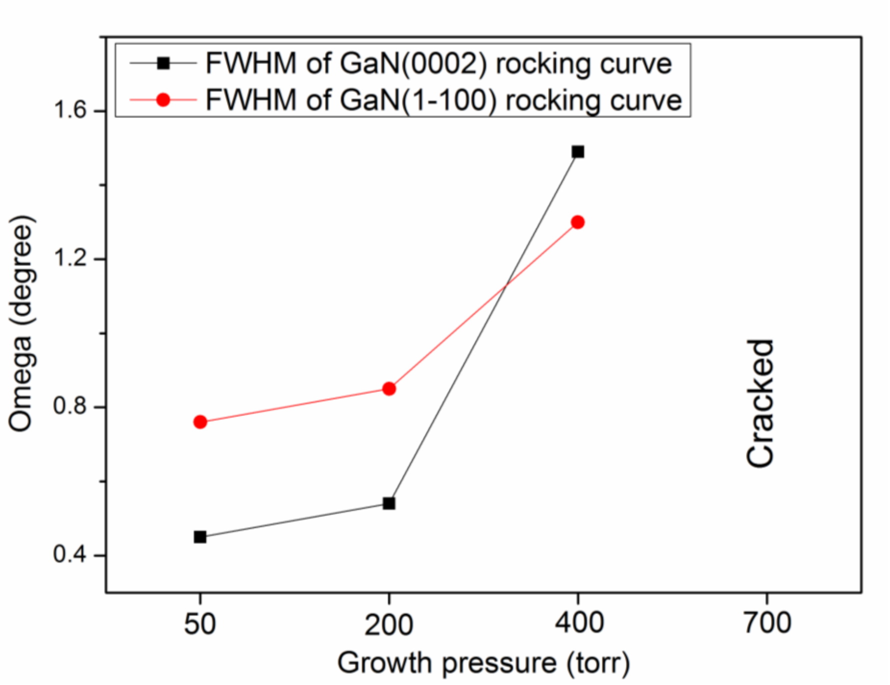

Supplementary Figure 10: The weight loss of $LiGaO_2$ and GaN single crystal in NH_3 (267 mbar) at different temperatures for 10 hours: (a) 900°C, (c) 950 ^{o}C and (e) 1000°C; The *ex situ* XRD of $LiGaO_2$ single crystal after nitridation in ammonia (267 mbar) for 10 hours: (b) 900°C, (d) 950 ^{o}C and (f) 1000°C.

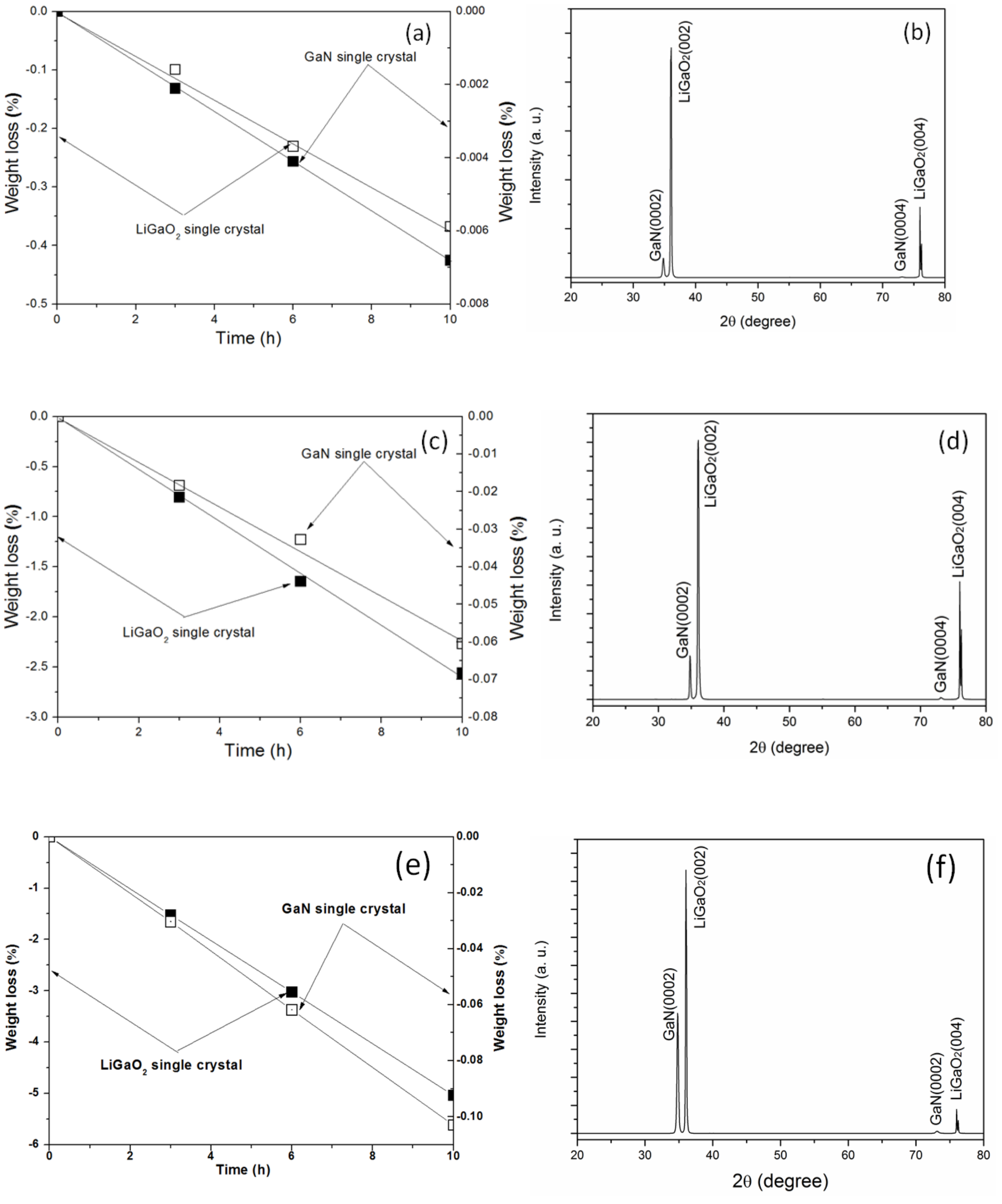

Supplement: Supplementary file 1 — Supplementary Information [file 41467_2017_2197_MOESM1_ESM.pdf]
